# Collateral Sensitivity to β-Lactam Antibiotics in Evolved Apramycin-Resistant MRSA

**DOI:** 10.3390/ijms252212292

**Published:** 2024-11-15

**Authors:** Jingjing Wu, Shiqian Wu, Juan Liu, Changmin Li, Mei Zheng, Fuhao Li, Yan Zhang, Yashuang Wu, Yang Yu

**Affiliations:** 1 State Key Laboratory for Animal Disease Control and Prevention, South China Agricultural University, Guangzhou 510642, China; jingjingwu789@163.com (J.W.); wsq19991027@163.com (S.W.); liuxiaojuan0505@163.com (J.L.); 15738737393@163.com (C.L.); mmzhengmei@gzucm.edu.cn (M.Z.); lifuhao857@163.com (F.L.); b20233050453@cau.edu.cn (Y.Z.); 15638250987@163.com (Y.W.); 2 National Risk Assessment Laboratory for Antimicrobial Resistance of Animal Original Bacteria, South China Agricultural University, Guangzhou 510642, China; 3 Guangdong Wenshi Dahuanong Biotechnology Co., Ltd., Yunfu 527400, China; 4 Animal Laboratory Center, Guangzhou University of Chinese Medicine, Guangzhou 510405, China

**Keywords:** collateral sensitivity, methicillin-resistant *Staphylococcus aureus*, apramycin, β-lactam antibiotics

## Abstract

Collateral sensitivity is an evolutionary trade-off for bacteria where acquiring resistance to one antibiotic results in an increased sensitivity to another antibiotic. This study was designed to evaluate the collateral sensitivity of methicillin-resistant *Staphylococcus aureus* (MRSA) to β-lactam antibiotics induced by the evolution of resistance to apramycin. Collateral sensitivity to ampicillin, cephazolin, ceftriaxone, cefotaxime, cefepime and cefquinome occurred after MRSA were exposed to apramycin and induced to acquire resistance. This sensitivity was associated with reduced β-lactamase activity and decreased expression of the *mecA* gene. We also found a decrease in the proton motive force and decreased efflux activity. These results provide new insights into collateral sensitivity-based strategies for the treatment of MRSA.

## 1. Introduction

Methicillin-resistant *Staphylococcus aureus* (MRSA) is typically transmitted via food and animals, and these skin and bloodstream infections pose a threat to the animal breeding industry and public health [1,2]. Long-term treatment is required for *S. aureus* infections but resistance resulting from long-term antibiotic use can lead to many complications [3,4]. For instance, MRSA bacteremia is associated with increased total hospitalization costs, prolonged hospital stays and higher in-hospital mortality, and these increase the burden of healthcare on the community and the financial pressure on individuals [5,6,7]. MRSA are resistant to a wide range of antibiotics and, when coupled with the ability to evade host immunity within biofilms, this additionally promotes its pathogenicity [8,9,10].

Current MRSA control programs include antimicrobial photodynamic therapy [11], bacteriophage therapy [12] and combination therapies that have provided some successes in clinical MRSA treatment [13]. In particular, combination therapy has emerged as the most prevalent strategy, and daptomycin-β-lactam and vancomycin-β-lactam combinations have been highly effective treatments [14,15]. However, the major obstacle to the success of these treatments has been selection pressure from increased antibiotic use that contributes to resistance development [16]. The acquisition of resistance is also linked to host fitness costs and collateral sensitivity to other drugs can occur during the evolution of resistance [17,18]. Collateral sensitivity is thus an evolutionary trade-off and currently cannot be predicted accurately [19]. For example, mutations in the *trkH* gene encoding a low affinity K^+^ transporter protein in aminoglycoside-resistant *Escherichia coli* resulted in increased sensitivity to β-lactams, tetracycline and chloramphenicol [20]. Recently, researchers have found that utilizing combination therapy based on collateral sensitivity therapies in carbapenem-resistant *Klebsiella pneumoniae* (CRKP) was able to limit the evolution of resistance to CRKP in vivo and in vitro [21]. Therefore, collateral sensitivity-directed antibiotic combinations are not only effective in treating bacterial infections but also have the potential value of slowing down the evolution of drug resistance [22]. Therefore, this strategy is expected to be important in MRSA treatment and slowing the evolution of resistance.

Apramycin was isolated in 1967 from *Streptomyces tenebrarius* obtained from soil samples in Sonora, Mexico [23,24]. Due to its unique chemical structure, apramycin is less toxic compared to other aminoglycosides [25]. Several in vitro studies have demonstrated the strong antibacterial activity of apramycin against highly resistant *Acinetobacter baumannii*, *E. coli*, *Klebsiella pneumoniae*, *Enterobacter ales* and Gram-positive bacteria. In the case of methicillin-susceptible *S. aureus*, MRSA and vancomycin intermediate-resistant *S. aureus*, apramycin has shown consistent in vitro bactericidal activity [26,27,28]. Apramycin has also been tested in combinations with meropenem and these have displayed efficient synergistic effects in vitro [29]. Therefore, the search for combinations of apramycin with other antibiotics on the basis of collateral sensitivity may be of great value for the clinical management of MRSA as well as for public health safety.

In this study, we evaluated the collateral sensitivity between apramycin-resistant MRSA and other antibiotics. We then analyzed the evolutionary trait of collateral sensitivity occurring in MRSA to detect fundamental changes in resistant strains. Finally, we further analyzed genomic data to explore possible mechanisms for this trade-off.

## 2. Results

### 2.1. MIC Determinations of MRSA Clinical Isolates

We initially assessed the MICs for apramycin in 112 MRSA isolates, and an epidemiological cut-off value of 32 mg/L was assigned as the breakpoint to distinguish between resistance and susceptibility [28,30]. The MIC_50_ was 4 mg/L and the MIC_90_ was 8 mg/L. Overall, 98.21% (110/112) of the MRSA isolates were susceptible to apramycin and 1.79% (2/112) of the isolates were resistant to apramycin. The MIC results indicated in vitro antimicrobial activity of apramycin against MRSA strains (Supplementary Materials S1).

### 2.2. Experimental Induction of Apramycin Resistance

**Figure 1:**
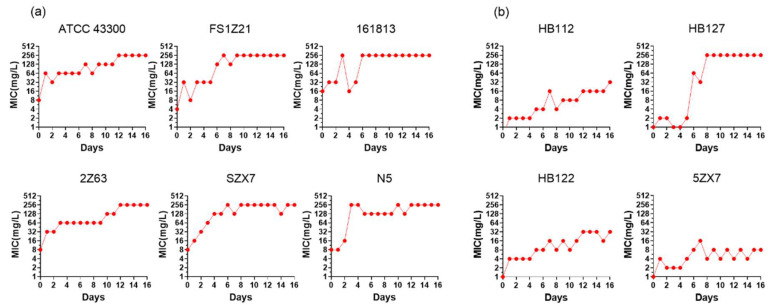
Effects of apramycin on the development of antibiotic resistance in MRSA. MICs for apramycin were determined daily. (**a**) Six strains rapidly developed resistance to apramycin within three days (MIC > 32 mg/L). (**b**) Four strains remained sensitive to apramycin over seven days.

*S. aureus* ATCC 43300 and nine randomly selected apramycin-sensitive MRSA isolates developed resistance under sustained selective pressure at progressively increasing concentrations of apramycin. After 16 continuous days of exposure, all strains except strain 5ZX7 became resistant to apramycin. The MIC values increased 16- to 64-fold. Six strains rapidly developed resistance to apramycin (MIC > 32 mg/L) within three days (Figure 1a). The other four strains developed resistance more slowly and remained sensitive to apramycin after seven days. Notably, the MICs of isolates HB122, HB112 and 5ZX7 increased 2- to 16-fold but did not exceed 32 mg/L and resistance persisted after three generations of culture in a drug-free control medium (Figure 1b).

### 2.3. Collateral Sensitivity Between Apramycin and β-Lactams

**Figure 2:**
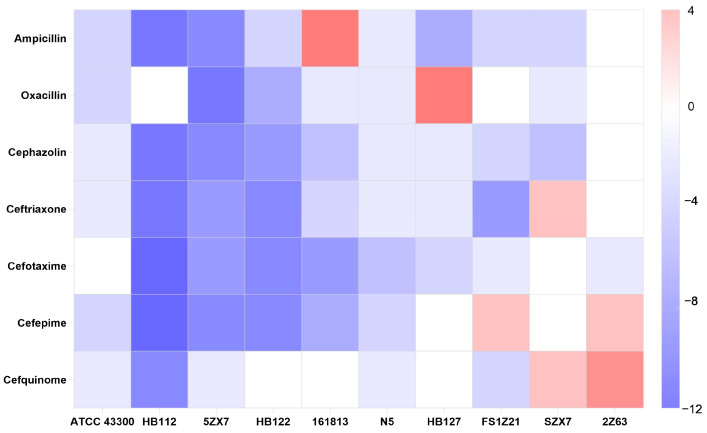
Sensitivity of apramycin-resistant mutants to β-lactams. Fold change in MIC values for each antibiotic are represented as blocks. Values are listed as log_2_.

We also determined the β-lactam sensitivities of the abovementioned test isolates, and most apramycin-evolved strains displayed an increased susceptibility to β-lactams (Table 1). A heat map was created to represent the fold change in the MIC of apramycin-evolved strains against β-lactam antibiotics, emphasizing cross-resistance and collateral sensitivity. Strains ATCC 43300, HB112, 5ZX7, HB122, 161813, N5 and HB127 showed collateral sensitivity with a 2- to 256-fold reduction in the MIC against most of the selected β-lactam antibiotics. These results indicated that the evolution of apramycin resistance was accompanied by collateral sensitivity between apramycin and β-lactam antibiotics. In addition, strain HB127 was found to be cross-resistant to oxacillin, strain FS1Z21 was cross-resistant to cefepime, strain SZX7 was cross-resistant to cefquinome and ceftriaxone and strain 2Z63 was cross-resistant to cefquinome and cefepime (Figure 2).

### 2.4. Collateral Sensitivity Associated with a Fitness Cost

**Figure 3:**
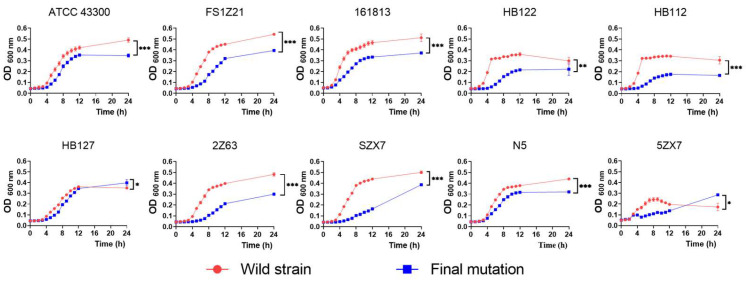
Bacterial growth curves. Effect of apramycin resistance on in vitro growth of *S. aureus* isolates. The graphs depict growth of 10 susceptible (red dots) and 10 apramycin-resistant (blue squares) clinical MRSA isolates followed over 24 h at 37 °C in LB broth. Each data point shown is the mean (±SD) for three independent experiments. Significant differences were assessed by one-way analysis of variance: *: *p* < 0.05; **: *p* < 0.01; ***: *p* < 0.001.

**Figure 4:**
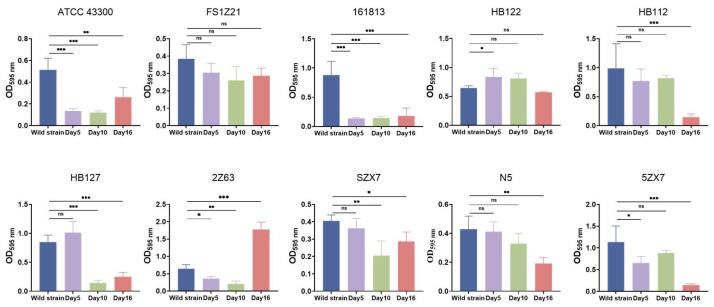
Biofilm formation of parental and evolved MRSA strains. Strains were evolved and sampled on days 5 and 16. For reference, biofilm formation data from isolates with no drug exposure were used as comparison (first bar on each graph). Each data point shown is the mean ± SD of three independent experiments. Significant differences were assessed by one-way analysis of variance: ns: not significant; *: *p* < 0.05; **: *p* < 0.01; ***: *p* < 0.001.

Growth rate testing of our experimental strains indicated that parental strains exhibited higher growth rates compared to their corresponding resistant strains (Figure 3). We also found significant differences in biofilm formation capacity between parental and pre- and post-evolved apramycin-resistant strains. With the exception of strain 2Z63 and strain HB122, all 16-day strains exposed to apramycin exhibited compromised biofilm formation compared to the unexposed control strains. Strains were also selected on days 5 and 10 of the evolution cultures and 80% of these strains exhibited decreased biofilm formation capacity at both times (Figure 4). Strain morphologies also differed when compared with those lacking drug exposure. The resistant strains exhibited significantly smaller colony morphologies compared to the parental strains, suggesting at least one cost of resistance development was reduced colony size (Supplementary Materials S2). Together, we found that β-lactam collateral sensitivity was accompanied by reduced growth, impaired biofilm formation and diminished colony size.

**Figure 5:**
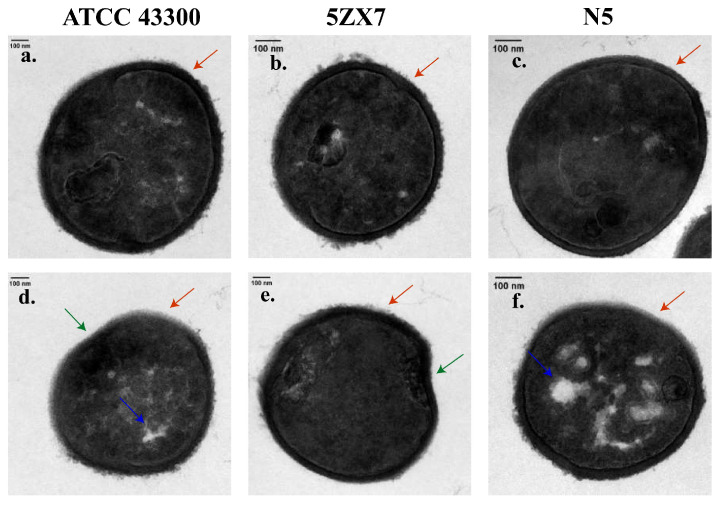
Transmission electron microscopy cross-section of ((**a**) ATCC 43300, (**b**) 5ZX7, (**c**) N5) parental strain cells and ((**d**) ATCC 43300, (**e**) 5ZX7, (**f**) N5) apramycin-evolved strain cells. Red arrows indicate loss of cell wall density. Green arrows indicate deformation of shape of *S. aureus* cells. Blue arrows indicate reduction of cytoplasm. Scale bars represent 100 nm.

To obtain additional data concerning apramycin-evolved strains’ impact on S. aureus cell morphology, cross-sections of bacterial cells (parental strains and apramycin-evolved strains) were performed using transmission electron microscopy. In Figure 5, representative pictures of three S. aureus strain cells are presented. Pictures a, b and c of Figure 5 show bacterial cells are parental strain cells, with an oval shape typical of S. aureus, whereas the apramycin-evolved strain cells present deformed morphology, particularly visible in pictures d,e of Figure 5. The deformation pattern included sunken cells (loss of oval shape, green arrows in Figure 5d,e). As seen in Figure 5a,b, the walls of the parental strain cells are evenly contrasted and strongly distinguished from the cytoplasm, while apramycin-evolved strain cells have a lower contrast that can be observed between the cytoplasm and the cell wall (marked with red arrows in Figure 5), indicating a loss of wall density. In the case of apramycin-evolved strains, reduction of the cytoplasm is also visible (blue arrows in Figure 5), suggesting lower cytoplasm density.

### 2.5. Evaluation of β-Lactamase Activity

**Figure 6:**
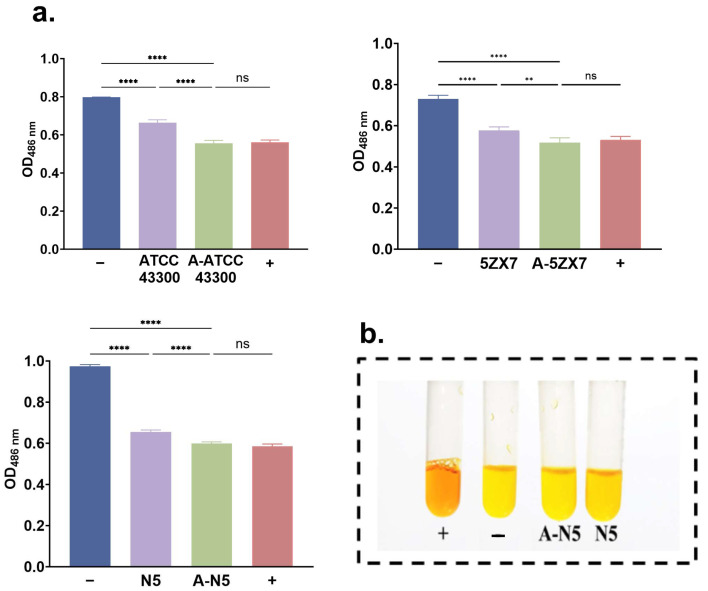
β-Lactamase activity. (**a**) Comparison of three parental strains and apramycin-evolved strain designated with the prefix A. (**b**) Color change of β-lactamase produced in group N5. + is positive control containing 1 mg/mL of sulbactam; − is negative control containing 2% Tween 80. Each data point shown is the mean ± SD for three independent experiments. Significant differences were assessed by one-way analysis of variance: ns: not significant; **: *p* < 0.01; ****: *p* < 0.0001.

β-lactamase activity was measured in culture supernatants as well as by a nitrofuran quantification assay. We found a decrease in enzyme activity in ATCC 43300, N5 and 5ZX7 that was similar to the sulbactam positive control (Figure 6a). Visual inspection of the color of the supernatant demonstrated that the resistant strain A-N5 was lighter in color than the parental strain N5, and this was consistent with the β-lactamase results (Figure 6b).

### 2.6. Relative Expression of mecA

**Figure 7:**
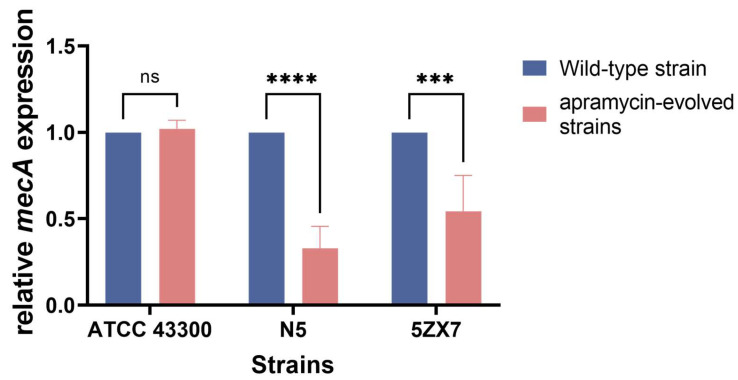
Expression of *mecA*. Fold change in gene expression normalized to 16S rRNA levels and relative to the untreated control. Values represent the mean ± SD for three independent experiments. Significant differences were assessed by one-way analysis of variance: ns: not significant; ***: *p* < 0.001; ****: *p* < 0.0001.

Expression of the *mecA* gene is associated with β-lactam resistance, and we found it was downregulated in 66.67% of strains compared to the parental counterparts. In particular, *mecA* levels were significantly lower in strains N5 and 5ZX7 but remained almost unchanged in the control ATCC 43300 strain (Figure 7).

### 2.7. Resistance in Evolved Strains Influences Proton Motive Force and Efflux Pumps

**Figure 8:**
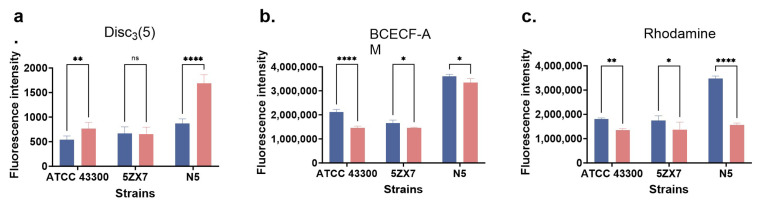
Efflux pump activity. Blue and red bars represent parental and induced evolutionary endpoint strains, respectively. (**a**) Bacterial membrane potential energy (Δψ) before and after the evolution of drug resistance. (**b**) Transmembrane proton gradient before and after the evolution of drug resistance (ΔpH). (**c**) Efflux functions before and after the evolution of drug resistance. Values represent the mean ± SD for three independent experiments. Significant differences were assessed by one-way analysis of variance: ns: not significant; *: *p* < 0.05; **: *p* < 0.01; ****: *p* < 0.0001.

We additionally determined whether collateral sensitivity, i.e., *mecA* resistance, was related to efflux pump activity. We evaluated the ΔpH and Δψ of the cultures since these are two key components of the proton motive force (PMF) needed to generate ATP [32].We utilized the pH indicator BCECF-AM and the dye Disc_3_(5) for these assays, respectively. The evolved mutants displayed significant reductions in fluorescence intensity for these dyes, indicating that they had decreased or dissipated the pH gradient and disrupted the PMF (Figure 8b). In addition, the amount of membrane potential fluorescence decreased in the resistant bacteria compared with the parentals, indicating that the final mutations significantly dissipated membrane potential (Δψ) in 2/3 strains (Figure 8a). We additionally examined efflux capacity using rhodamine efflux assays. The rhodamine fluorescence intensity of the resistant strains was lower than the parental strain in all three isolates and this linked efflux capacity with resistance (Figure 8c).

## 3. Discussion

The emergence of MRSA infections has posed a significant challenge for human use of β-lactams [33]. As a potential solution, the beneficial evolutionary trade-off of using collateral sensitivity to reverse antibiotic resistance may be a viable alternative [34]. A deeper understanding of the evolutionary processes linked to apramycin resistance could offer valuable insights for future treatment strategies. The essence of collateral sensitivity lies in bacteria’s adaptive and evolutionary responses to drugs, ensuring their survival and reproduction. The main mechanisms include the following: (1) increased drug absorption, meaning the enhanced permeability of the cell membrane or reduced efflux of antibiotics; (2) chemical effects, such as the activation of drugs through specific proteins, resulting in the formation of more chemically active substances; (3) cellular effects, such as changes in cellular functions or regulatory pathways that indirectly enhance the effectiveness of antibiotics; and (4) target effects, meaning the increased binding of antibiotics to modified targets. Furthermore, the mechanisms of collateral sensitivity may be related to chromosomal mutations, the acquisition of resistance plasmids and the generation of resistance genes [35]. Although the collateral sensitivity of β-lactams and aminoglycosides has been extensively studied [36,37,38,39], little is known about the evolution and mechanism of apramycin resistance. Our study explored the evolution of apramycin resistance and examined the potential reversal of MRSA resistance to β-lactams.

In the current study, we examined 10 MRSA strains that demonstrated the rapid and stable development of apramycin resistance when exposed to an antibiotic. While apramycin holds promise for antibacterial treatment in the future, careful attention to the duration of administration, precise dosage control and regimen management is warranted. The evolution of drug-resistant bacteria often alters growth and reproductive capacity under antibiotic pressure, leading to differences in physiological characteristics between parental strains and evolved strains [40,41]. *S. aureus*, a pathogen, may employ various strategies to resist antibiotic treatment. One such strategy is the formation of small colony variants (SCVs), which are naturally occurring, slowly growing subpopulations with unique phenotypic characteristics and pathogenic traits [42]. In this study, we observed that bacteria that had evolved resistance showed a decrease in colony numbers and smaller colony sizes under the same conditions. This phenomenon, distinct from SCVs, is referred to as the fitness cost of bacterial resistance [40,41]. The bacteria that developed resistance also exhibited reduced growth rates, decreased final cell masses and altered colony morphologies after the removal of the corresponding drugs. Moreover, we observed impairments in biofilm formation and changes in the cell wall and in the cytoplasm. Cells are protected from lysis in low osmotic atmospheres owing to the high tensile strength of the cell wall, and such damage to the cell wall certainly decreases the tolerance of the cells to antibiotic pressures. Since biofilms act as barriers that hinder antibiotic diffusion into bacterial cells, they can serve as indicators of the sensitivity of resistant strains to antibiotics [43]. Notably, 90% of the drug-resistant strains showed evident deficiencies in biofilm formation, signaling a decrease in their ability to resist antibiotics, which could be advantageous for drug therapy.

The rapid development of apramycin resistance in clinical bacterial populations indicated an urgent demand for a strategy to repress resistance development before large-scale clinical use. Our exploration of apramycin resistance in MRSA revealed the acquisition of apramycin resistance simultaneously conferred an increased sensitivity to β-lactams after prolonged apramycin exposure. The β-lactams ampicillin, cephazolin, ceftriaxone, cefotaxime, cefepime, cefquinome and oxacillin exhibited varying degrees of drug susceptibility when acting on the same resistant strain. It is worth noting that although oxacillin demonstrates a decrease in MIC, based on these susceptibility breakpoints, only three of the apramycin-resistant derivatives (HB122, HB112 and 5ZX7) became oxacillin-susceptible. This suggested that the occurrence of collateral sensitivity may be related to the site of drug action and antimicrobial spectrum. It is essential to uncover the underlying mechanism of apramycin resistance with collateral sensitivity to β-lactams.

The *mecA* (methicillin resistance) gene is located on a mobile chromosomal genetic island and encodes PBP2a that has a low affinity to most β-lactams and is a major determinant of MRSA resistance [44]. The regulatory systems encoded by the *blaZ* or *mecA* gene clusters sense the presence of β-lactam antibiotics through membrane-embedded sensor-inducer proteins, such as BlaR1 or MecR1. Upon sensing the antibiotic, a signal is triggered that activates the cleavage of transcriptional repressors like BlaI or MecI in the cytoplasm, relieving repression of the *blaZ* and *mecA* genes [45,46,47]. We found that an increased sensitivity of MRSA to apramycin corresponded with decreased *mecA* expression, suggesting a potential weakening of PBP2a function [48]. Our drug-resistant test strains evolved to express decreased β-lactamase activity compared to the parental strains so that collateral sensitivity was due to a reduction in the expression level of the *mecA* gene. However, in the ATCC43300 strain, we did not observe a significant change in *mecA* gene down-regulation. We believe that collateral sensitivity may be related to different mechanisms rather than arising from a single mechanism.

Collateral sensitivity between aminoglycosides and β-lactams depends on the bacterial PMF that represents an energy channel located on the bacterial cell membrane necessary for ATP production [49,50]. The PMF maintains the electrochemical proton gradient of the entire cell membrane that is determined by the transmembrane proton gradient (ΔpH) and transmembrane potential (ΔΨ) [51]. Changes in ΔpH may alter the antibiotic efflux system and proton dynamics and thus alter antibiotic resistance [52]. Our results indicated changes in ΔpH and ΔΨ in our evolved strains and demonstrated that collateral sensitivity was linked to the PMF that could be related to altered ΔpH or ΔΨ levels. In turn, this was also directly related to drug efflux pump activity and is consistent with a decrease in rhodamine efflux assay results as presented above. Intracellular drug levels are largely determined by efflux rates [53] so that the PMF disruption in MRSA under continuous exposure to apramycin led to reduced activity that in turn altered β-lactam sensitivity. This finding has been corroborated in other strains where the disruption of PMF led to decreased activity of the drug efflux pump system following aminoglycoside resistance [20].

The *apmA* gene, which encodes an acetyltransferase known to confer resistance to apramycin, particularly in veterinary isolates, could influence our research findings [54]. Its presence may interact with other resistance mechanisms observed in our study, potentially leading to altered sensitivities to apramycin and other antibiotics. To investigate this, we conducted PCR experiments on 10 MRSA strains, including *apmA*-positive controls. The unpublished observations revealed that none of the 10 MRSA strains contained the *apmA* gene.

We recognize the limitations in our investigation of the genetic and molecular changes underlying the phenotypic differences in MRSA strains. In the future, more in-depth studies in transcriptomics and proteomics are essential to enhance our understanding of the complex interactions involved, ultimately leading to more effective strategies for managing antibiotic resistance.

This study provides compelling evidence that MRSA can readily attain high levels of apramycin resistance in vitro that is accompanied by fitness costs such as slow growth and defects in biofilm formation. Furthermore, this alters apramycin resistance and collateral sensitivity to β-lactams linked to reduced *mecA* gene expression, decreased β-lactamase activity and PMF alterations that affect efflux pump activity. These results explain the molecular *mecA* mechanism of resistance evolution and collateral sensitivity of apramycin and β-lactams and lay a foundation for developing effective regimens and combination therapies against MRSA. These results also provide a proof-of-concept that can serve as a guide to reverse β-lactam antibiotic resistance in MRSA.

## 4. Methods and Materials

### 4.1. Bacterial Strains and Background Information

A total of 112 MRSA strains were isolated from pigs, chickens and ducks in 10 different regions of China and from clinical patients in two different hospitals in Guangzhou, China, during 2011–2016 [55]. *S. aureus* standard strain ATCC 29213, *Escherichia coli* standard strain ATCC 25922 and methicillin-resistant *S. aureus* standard strain ATCC 43300 were purchased from Guangdong Provincial Microbial Strain Collection Center (Guangzhou, China). All MRSA isolates were confirmed by MALDI-TOF/MS system (Shimadzu-Biotech, Kyoto, Japan), multiplex PCR amplification and DNA sequencing of the *mecA* gene.

### 4.2. Antimicrobial Susceptibility Tests

Minimum inhibitory concentration (MIC) testing of antibiotics were performed using a standard broth microdilution method according to the CLSI 2024 guidelines. Breakpoints were identified using CLSI for categorical interpretation of susceptibility and resistance to selected antibiotics in this study [56]. All antimicrobial susceptibility testing was quality controlled against *E. coli* ATCC 25922 and *S. aureus* ATCC 29213. Briefly, single bacterial colonies were incubated in Mueller–Hinton broth (MHB) at 180 rpm for 4–5 h at 37 °C. Subsequently, in a clear UV-sterilized 96-well microtiter plate, two-fold dilution series of antibiotics in MHB was mixed with an equal volume of bacterial suspension and incubated at 37 °C for 16–20 h before reading MIC values. The MIC was determined as the lowest concentration of antimicrobial agents visibly inhibiting microbial growth.

### 4.3. In Vitro Induction of Apramycin Resistance

Apramycin-resistant strains were generated from 10 clinical MRSA isolates randomly selected from our group of MRSA isolates by exposure to two-fold increasing concentrations of apramycin during a 16-day continuous passaging procedure. Briefly, overnight cultures of strains were diluted to 10^6^ CFU/mL and exposed to apramycin (0.125 to 256 mg/L) for 16–18 h at 37 °C. The antibiotic dilution containing the last visible trace of bacterial growth were mixed with the MIC wells and again exposed to increased apramycin as described above. The abovementioned procedures were repeated for 16 days, and MICs were determined against the induced strains daily to monitor the development of apramycin susceptibility. The induced apramycin-resistant strains were passaged for three generations in control medium without any drug and then the MIC was determined to confirm the acquisition of stable resistance.

### 4.4. Turbidimetric Assays

A multifunctional plate reader (Beckman, Hercules, CA, USA) was used for the analysis of culture turbidity. Single colonies were incubated in LB broth at 180 rpm at 37 °C overnight and then 200 µL of diluted bacterial solution was added to the wells of a 96-well plate. The value of optical density at 600 nm to measure culture density was recorded and data were graphed using GraphPad Prism 8 (Boston, MA, USA) for further analysis.

### 4.5. Biofilm Assays

Briefly, the strains were grown overnight in LB broth, washed with PBS and then inoculated into BHI broth at a 1:100 dilution. The samples were dispensed into 96-well plates and incubated at 37 °C for the indicated times. The plates were then washed twice with 0.9% NaCl solution. After incubation with 96% ethanol for 15 min, the plates were stained with 0.1% crystal violet (CV) for 20 min. Subsequently, the plates were washed four times with water, the stain was dissolved with 30% acetic acid and the samples were transferred to new microtiter plates. The ability of biofilm to form was measured at absorbance of 595 nm.

### 4.6. Morphological Analysis by Transmission Electron Microscopy (TEM)

Bacteria were prepared for TEM by centrifugation at low speed, and the pellets were added to a pre-cooled 4% glutaraldehyde solution and fixed at 4 °C overnight. Samples were then washed 3× with 0.1 M phosphate buffer (pH 7.4) and then fixed in 1% osmium tetroxide solution for 2 h. Bacteria were harvested using a hole filter, scraped off and the collection was dispersed into tempered agarose that was allowed to gel at room temperature and then was cut into cubes. After post-fixation of the cubes in 2% hydrogen peroxide acetate for 2 h, the cubes were washed 3× for 20 min each using purified water. Dehydration was performed by incremental concentrations of isopropanol: 50, 70 and 90% × 10 min each and 100% for 2 × 15 min. The cubes were permeated with LR white acrylic resin (Electron Microscopy Services, Hatfield, PA, USA). Sections were made on an ultrathin microtome using a glass knife and then stained with uranyl acetate and lead citrate and visualized using a Talos L120C transmission electron microscope (FEI, Hillsboro, OR, USA).

### 4.7. Evaluation of β-Lactamase Activity Using the Nitrocefin Test

We used a colorimetric assay to determine β-lactamase activity. The colorimetric assay is based on the hydrolysis of the chromogenic nitrocefin that produces colored products proportional to β-lactamase activity. Measurement of the absorbance of the colored product at 486 nm therefore correlates with β-lactamase activity. Oxacillin sodium monohydrate was added to the bacterial solution with an OD_600_ = 0.5, and the suspension was collected by centrifugation at 3000 rpm for 15 min after incubation. The obtained MRSA cells were washed 3× and then ultrasonicated. A portion of the supernatant (2 mL) was taken and incubated at 37 °C for 30 min to which 200 μL nitrocefin (0.5 mg/mL) was added. The absorbance of the mixture was measured at 486 nm after 10 min of incubation. β-lactamase activity was monitored by measuring the increase in absorbance at 486 nm.

### 4.8. DiSC3 (5) Assays

Overnight cultures of bacterial strains used for membrane permeability (see above) were washed with PBS and suspended to OD_600_ nm = 0.5. The DiSC_3_(5) probe was added to 0.5 μM, and 200 μL of cells were collected and incubated in the dark for 30 min. The change in membrane potential energy was measured using excitation/emission wavelengths of 622/670 nm.

### 4.9. ROS and ΔpH Measurements

Reactive oxygen species (ROS) were measured in the presence of the fluorescent probe 2′7-dichlorofluorescein diacetate (DCFH-DA) (10 μM). Briefly, overnight cultures of the abovementioned strains used for membrane permeability were washed 3× with 0.01 M phosphate buffer (pH 7.4) and suspended to OD_600_ nm = 0.5, and DCFH-DA was then added to 10 μM and the mixture was incubated at 37 °C for 30 min. The cells were then washed 3× with phosphate buffer and 190 μL of probe-labeled bacteria were added to a 96-well plate. The fluorescence intensity was measured at excitation/emission wavelengths of 488/525 nm.

The fluorescent probe BCECF-AM was used to assess the ΔpH of the bacteria using 1 × 10^8^ CFU/mL in phosphate buffer as per above. BCECF-AM was added to 3 μM and incubated in the dark for 30 min, and 200 μL was added to black 96-well plates and fluorescence intensity was measured at 490/530 nm excitation/emission wavelengths as per above.

### 4.10. qRT-PCR Assays

Strains N5, 5ZX7 and ATCC 43300 and an apramycin-resistant strain were incubated at 37 °C and 180 rpm for 4–5 h to obtain exponential-phase bacteria. Total RNA was extracted using a commercial kit (Omega Bio-Tek, Norwalk, GA, USA) and quantified using UV spectroscopy in a Nanodrop spectrophotometer (Thermo Scientific, Pittsburg, PA, USA). qRT-PCR was performed using the Taq Pro Universal SYBR qPCR Master Mix kit (Vazyme Biotech, Nanjing, China). Cycling conditions consisted of the following: 95 °C for 30 s followed by 40 cycles of 10 s at 95 °C and 30 s at 60 °C. All samples were analyzed in triplicate and the gene 16S rRNA was used as an endogenous control. Fold changes in gene expression were analyzed by the 2^−ΔΔCT^ method, and GraphPad Prism 8 was used for graphing. The primers used in this study are listed in Table 2 below.

## Figures and Tables

**Table 1:** MICs of parental strains and apramycin-resistant isolates. AP: apramycin (cut-off ≥ 32 mg/L); CEQ: cefquinome (cut-off ≥ 1 mg/L) [31]; AMP: ampicillin (cut-off ≥ 0.5 mg/L); CZ: cephazolin (cut-off ≥ 32 mg/L); CTR: ceftriaxone (cut-off ≥ 64 mg/L); CTX: cefotaxime (cut-off ≥ 64 mg/L); CPM: cefepime (cut-off ≥ 32 mg/L); OXA: oxacillin (cut-off ≥ 4 mg/L). W: parental strains; A: apramycin-evolved strains; F: fold-change of MIC.

Strains	MIC (mg/L)																							
	AP			CEQ			AMP			CZ			CTR			CTX			CPM			OXA		
	W	A	F	W	A	F	W	A	F	W	A	F	W	A	F	W	A	F	W	A	F	W	A	F
N5	8	256	32	8	4	−2	128	64	−2	2	1	−2	16	8	−2	32	4	−8	16	4	−4	64	32	−2
FS1Z21	4	256	64	8	2	−4	64	16	−4	2	0.5	−4	32	1	−32	8	4	−2	8	16	2	64	64	0
161813	16	256	16	32	32	0	32	256	8	256	32	−8	256	64	−4	256	8	−32	256	16	−16	256	128	−2
HB127	16	256	16	2	2	0	256	16	−16	2	1	−2	8	4	−2	8	2	−4	8	8	0	8	64	8
HB122	1	32	32	2	2	0	2	0.5	−4	32	1	−32	256	4	−64	256	4	−64	64	1	−64	16	1	−16
SZX7	16	256	16	16	32	2	256	64	−4	32	4	−8	128	256	2	32	32	0	32	32	0	64	32	−2
2Z63	8	256	32	32	128	4	256	256	0	64	64	0	256	256	0	64	32	−2	64	128	2	128	128	0
HB112	0.5	32	64	32	0.5	−64	64	0.5	−128	256	2	−128	256	2	−128	256	1	−256	256	1	−256	0.5	0.5	0
ATCC 43300	8	256	32	4	2	−2	2	0.5	−4	2	1	−2	8	4	−2	2	2	0	4	1	−4	16	4	−4
5ZX7	1	8	8	2	1	−2	64	1	−64	128	2	−64	128	4	−32	128	4	−32	128	2	−64	128	1	−128

**Table 2:** Primers used in this study.

Gene	Primer
*mecA*	F: GGCCAATACAGGAACAGC
	R: GGAACGAAGGTATCATCTTGTAC
16S rRNA	F: GCTCGTGTCGTGAGATGTTGG
	R: TTTCGCTGCCCTTTGTATTGT

## Data Availability

Data are contained within the article and Supplementary Materials.

## Supplementary Materials

The following supporting information can be downloaded at: https://www.mdpi.com/article/10.3390/ijms252212292/s1.
