# Supplementary material for: Collateral Sensitivity to β-Lactam Antibiotics in Evolved Apramycin-Resistant MRSA
Source: Int J Mol Sci. 2024 Nov 15;25(22):12292. doi: 10.3390/ijms252212292 (PMC11594749; doi:10.3390/ijms252212292)
Supplement: Supplementary file 1 [file ijms-25-12292-s001.zip › ijms-3212137-supplementary.pdf]

| isolates | MIC(mg/L) of apramycin | | | | | | | | | | | $MIC_{50}$ | $MIC_{90}$ | Resistance (%) |
|---|---|---|---|---|---|---|---|---|---|---|---|---|---|---|
| | 0.25 | 0.5 | 1 | 2 | 4 | 8 | 16 | 32 | 64 | 128 | 256 | | | |
| all | 0 | 0 | 2 | 40 | 43 | 24 | 1 | 0 | 0 | 1 | 1 | 4 | 8 | 1.79 |

**Supplemental Material 1.** MIC distributions for apramycin for 112 MRSA isolates used in this study.

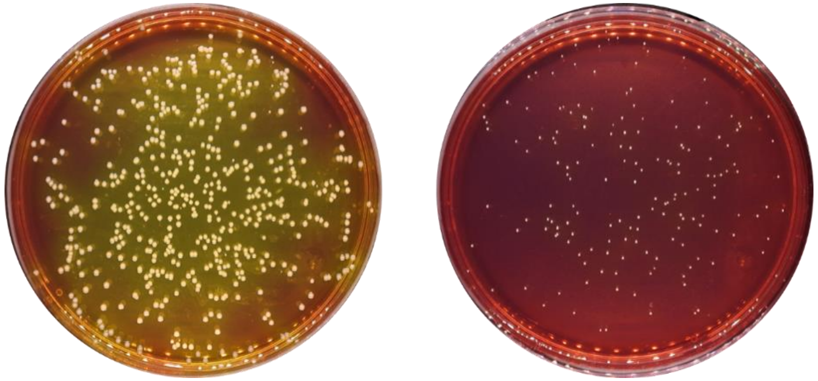

**Supplemental Material 2.** Colony morphology of the parental strain HB 112 (left) and its apramycin-evolved strain (right) grown in mannitol high-salt medium.
